# Treatment by Tuberculin

**Published:** 1914-05-09

**Authors:** 


### TREATMENT BY TUBERCULIN.

In our last issue appeared a summary of a sensational contribution by Dr. Noel Bardswell to the literature of tuberculin treatment for consumption. Those who are sufficiently interested to study the original report will find in it ample proof of the encomiums bestowed upon it by our contributor, and of the scientific and practical importance of the topic under discussion. They will find that, after many years of study and experiment with different varieties of tuberculin on the part of many eminent physicians, the most that Dr. Bardswell will say is that in the small minority of cases for which the perfected tuberculin treatment is thought to be justifiable it is doubtful whether it does much good and quite certain that it may do harm. This dubious conclusion will not, in all probability, be acceptable to the hyper-enthusiastic type of clinician, with whom the tuberculin treatment is a favourite because it allows him to "do something" for cases which more cautious colleagues have regarded as unsuitable for heroic measures. And to the laity it will be somewhat unnerving to hear such a very qualified verdict on a form of treatment which the bulk of the medical profession regards in a much more favourable light.

The explanation of the anomaly is not difficult. Tuberculosis is a disease so common that great authorities have declared that every civilised adult has suffered from at least one attack of it, though in most cases it has not been recognised as such. In a minority of individuals the invasion, instead of being spontaneously checked, develops to such a pitch that it causes recognisable symptoms. Even then recovery frequently ensues, often without, and more often with, such assistance as can be afforded by sanatorium treatment and other therapeutic measures. In more advanced cases the course of the disease is as a rule unfavourable, but even then periods of remission or of improvement, or of actual complete recovery, may supervene. So it comes to pass that the estimation of the value of any particular adjunct to treatment is a very complex business. The irregular unforeseeable course of the disease results in the ascription of any improvement, temporary or permanent, to the particular form of treatment in use at the time; and of any set-back to the inherently malign effects of the tuberculous processes. Hence the vast number of different treatments, each greatly vaunted by its discoverer and a select band of followers, which have been introduced in constant succession, only to be dropped as soon as they have been fully tested. There must have been two or three dozen such this century, most of them variations on the tuberculin theme. Whether any one of these numerous tuberculins, and methods of giving them, will eventually turn out to be of real help in the crusade against consumption Dr. Bardswell will not predict. All he says is that there is as yet no scientific proof that any of them do any good, and plenty of evidence that some of them have done a great deal of harm. There, till time has elapsed, the matter must be left, but it is reassuring to know that the problem is being further studied.

